# Supplementary material for: From sequence to enzyme mechanism using multi-label machine learning
Source: BMC Bioinformatics. 2014 May 19;15:150. doi: 10.1186/1471-2105-15-150 (PMC4229970; doi:10.1186/1471-2105-15-150)
Supplement: Additional file 2 — Java code of ml2db. Additional file ml2db_code.tar.gz contains the Java source code to run the multi-label machine learning experiments and save the results to database. The code’s Javadoc is included. [file 1471-2105-15-150-S2.zip › additional file 2/ml2db/ml2db/doc/main/ml2db/mulan/attributesfiltered/AttFilter.html]

AttFilter


JavaScript is disabled on your browser.


- Overview
- Package
- Class
- Use
- Tree
- Deprecated
- Index
- Help

- Prev Class
- Next Class

- Frames
- No Frames

- All Classes

- Summary:
- Nested |
- Field |
- Constr |
- Method

- Detail:
- Field |
- Constr |
- Method


main.ml2db.mulan.attributesfiltered

## Class AttFilter

- java.lang.Object
- - uk.ac.standrews.utils.main.Initialised
  - - main.ml2db.mulan.attributesfiltered.AttFilter

- All Implemented Interfaces:
  :   uk.ac.standrews.utils.main.Initialisable

  ---

    

  ```
  public class AttFilter
  extends uk.ac.standrews.utils.main.Initialised
  ```

  This class filters the attributes of a dataset (the test set) to make it
  compatible with another dataset (the training set). The result is a new
  dataset that can be used as test set for the specified training set.
  Main operations: 1. Attributes: only attributes present in the train set can
  appear in the test set (& all of them must be present) 2. Class attributes:
  all the classes of the train set \*and\* the test set must be conserved, to
  allow for train/test evaluation. This means that also the train arff must be
  regenerated to include all the classes present in the test set (the class
  numbering will change). 3. Mulan XML file (class labels): cannot be the same
  XML file of the training set, must also include all the classes in the test
  set. 4. New test ARFF File: the new test set arff file name should mention
  the original files and which attributes and classes were used for filtering
  (that is, left in the file or added to the file). 5. New train ARFF file
  name: (including also the test classes) 'trainset id' +
  'including\_classes\_of' + 'test\_set id' + '.arff' 6. New test ARFF record:
  when stored in the database, should contain trace (id/props file path) of the
  original training set and the "old" test set used to generate the "new",
  filtered, test set.

  Version:
  :   3 Sep 2010

  Author:
  :   Luna De Ferrari ldf(at)st-andrews.ac.uk

- - ### Field Summary

    - ### Fields inherited from class uk.ac.standrews.utils.main.Initialised

      `m_initialisationIsCorrect`
  - ### Constructor Summary

    Constructors

    Constructor and Description

    `AttFilter(int oldTrainArffId,
    int oldTestArffId,
    DbManagers dbMan)`
  - ### Method Summary

    Methods

    | Modifier and Type | Method and Description |
    | --- | --- |
    | `int` | `createNewArff(int oldArffRecordId)` |
    | `void` | `createNewTestArff()` |
    | `void` | `createNewTrainArff()` |
    | `ArffRecordsTable` | `getArffTable()` |
    | `DbManagers` | `getDbManagers()` |
    | `int` | `getNewTestArffId()` |
    | `int` | `getNewTrainArffId()` |
    | `int` | `getOldTestArffId()` |
    | `int` | `getOldTrainArffId()` |
    | `java.util.TreeSet<java.lang.String>` | `getTrainAttributesAndTrainTestClasses()` |
    | `java.lang.String` | `getTrainXmlPath()` |
    | `java.lang.String` | `getXmlPathById(int arffId)` |
    | `java.lang.String` | `getXmlQueryById(int arffId)` |
    | `static void` | `main(java.lang.String[] args)` |
    | `static void` | `manyFilterRuns()` Executes arff generation and filtering of several train-test files couples |
    | `static void` | `oneFilterRun()` |
    | `static int[]` | `run(int oldTrainArffId, int oldTestArffId, DbManagers dbMan)` Filter the old test arff by the train arff given and regenerate the old train set using all train+test classes. |
    | `void` | `writeFiles()` |

    - ### Methods inherited from class uk.ac.standrews.utils.main.Initialised

      `initialisationIsCorrect, setInitialisation, setInitialisationIsCorrect, setInitialisationNotCorrect`
    - ### Methods inherited from class java.lang.Object

      `equals, getClass, hashCode, notify, notifyAll, toString, wait, wait, wait`

- - ### Constructor Detail


    - #### AttFilter

      ```
      public AttFilter(int oldTrainArffId,
               int oldTestArffId,
               DbManagers dbMan)
      ```
  - ### Method Detail


    - #### createNewArff

      ```
      public int createNewArff(int oldArffRecordId)
      ```

      Parameters:
      :   `oldArffRecordId` - the database id of the old arff file

      Returns:
      :   the database id of the new, filtered arff file


    - #### createNewTestArff

      ```
      public void createNewTestArff()
      ```


    - #### createNewTrainArff

      ```
      public void createNewTrainArff()
      ```


    - #### getArffTable

      ```
      public ArffRecordsTable getArffTable()
      ```


    - #### getDbManagers

      ```
      public DbManagers getDbManagers()
      ```


    - #### getNewTestArffId

      ```
      public int getNewTestArffId()
      ```


    - #### getNewTrainArffId

      ```
      public int getNewTrainArffId()
      ```


    - #### getOldTestArffId

      ```
      public int getOldTestArffId()
      ```


    - #### getOldTrainArffId

      ```
      public int getOldTrainArffId()
      ```


    - #### getTrainAttributesAndTrainTestClasses

      ```
      public java.util.TreeSet<java.lang.String> getTrainAttributesAndTrainTestClasses()
      ```


    - #### getTrainXmlPath

      ```
      public java.lang.String getTrainXmlPath()
      ```


    - #### getXmlPathById

      ```
      public java.lang.String getXmlPathById(int arffId)
      ```


    - #### getXmlQueryById

      ```
      public java.lang.String getXmlQueryById(int arffId)
      ```


    - #### writeFiles

      ```
      public void writeFiles()
      ```


    - #### main

      ```
      public static void main(java.lang.String[] args)
      ```


    - #### manyFilterRuns

      ```
      public static void manyFilterRuns()
      ```

      Executes arff generation and filtering of several train-test files
      couples


    - #### oneFilterRun

      ```
      public static void oneFilterRun()
      ```


    - #### run

      ```
      public static int[] run(int oldTrainArffId,
              int oldTestArffId,
              DbManagers dbMan)
      ```

      Filter the old test arff by the train arff given and regenerate the old
      train set using all train+test classes.

      Parameters:
      :   `oldTrainArffId` - the record id of the original training arff
      :   `oldTestArffId` - the record id of the original test arff
      :   `resultsPropsFileName` - the database where to write the new arff records
      :   `dataDbPropsFile` - the database where to read the data to rebuild the arff files

      Returns:
      :   the identifiers of the new train and test arff records (in this
          order: train has index 0, test has index 1) in the database


- Overview
- Package
- Class
- Use
- Tree
- Deprecated
- Index
- Help

- Prev Class
- Next Class

- Frames
- No Frames

- All Classes

- Summary:
- Nested |
- Field |
- Constr |
- Method

- Detail:
- Field |
- Constr |
- Method
